# Effects of a Targeted Concurrent Training Program on the Exercise Adherence in Female Breast Cancer Survivors: A Randomized Controlled Trial

**DOI:** 10.3390/healthcare13040429

**Published:** 2025-02-17

**Authors:** Igor Herrero-Zapirain, Sergio Alvarez-Pardo, Arkaitz Castañeda-Babarro, Fabio García-Heras, Olga Pons-Llanas, Elena Oliete-Ramírez, Juan Mielgo-Ayuso

**Affiliations:** 1 Department of Health Sciences, Faculty of Health Sciences, University of Burgos, 09001 Burgos, Spain; igorjudo81@gmail.com (I.H.-Z.); jfmielgo@ubu.es (J.M.-A.); 2 Department of Sports, Isabel I University, 09003 Burgos, Spain; sergioal96@hotmail.com; 3 Health, Physical Activity and Sports Science Laboratory, Department of Physical Activity and Sports, Faculty of Psychology and Education, University of Deusto, 48007 Bilbao, Spain; 4 VALFIS Research Group, Department of Physical Education and Sports, Institute of Biomedicine (IBIOMED), University of León, 24071 Leon, Spain; fgarh@unileon.es; 5 Radiotherapy Department, La Fe University and Polytechnic Hospital, 46026 Valencia, Spain; pons_olg@gva.es; 6 Continuous Care Unit, Valencian Institute of Oncology Foundation, 46009 Valencia, Spain; eoliete@fivo.org

**Keywords:** physical activity adherence, breast cancer, concurrent exercise program

## Abstract

**Background/Objectives:** Increased time and adherence to physical activity, as well as increased intensity of physical activity, is one of the determining factors in improving survival and avoiding disease recurrence in female breast cancer survivors. The study aims to determine the effect of a 12-session concurrent training program on increasing moderate/vigorous physical activity time in this population. **Methods:** A total of (N = 72) female breast cancer survivors were randomized into an intervention group and a control group to perform a 12-session concurrent training program. The GPAQ questionnaire measured the time and intensity of physical activity and work they performed before starting the program and three months after completion of the program. **Results:** A total of (N = 15) women in the intervention group and (N = 22) women in the control group completed the questionnaire before and three months after completing the program. A significant increase in moderate, vigorous, and combined physical activity (PA) was observed in the intervention group (IG) in comparison to the control group (CG) at three months following the intervention. While these results are promising, it is important to note that the observed improvements in PA adherence reflect associations rather than direct causal relationships. While these changes are statistically significant, they also reflect meaningful improvements in clinical outcomes. A notable finding was the significant decrease in sedentary time observed in the IG, which is likely to have contributed to the observed improvement in adherence. **Conclusions:** Concurrent training shows an increment in the time and intensity of daily physical activity performed by breast cancer survivors, which is a determining aspect of the survival and non-relapse of the disease in this population.

## 1. Introduction

Breast cancer (BC) is a leading cause of mortality worldwide and the most prevalent form of carcinoma. Its incidence has been steadily rising over the last two decades [1,2]. Patients with BC frequently experience significant musculoskeletal changes, including osteosarcopenia, characterised by the loss of muscle mass and bone density. These changes are directly correlated with lower survival rates and increased cancer recurrence [1,3]. Sarcopenia, in particular, has been identified as a prognostic factor for mortality in both metastatic and non-metastatic BC patients [1]. Additionally, low muscle mass is associated with increased frailty, dependency, reduced autonomy, and lower bone mineral density [3].

Concurrent training, when properly and safely planned, is an effective intervention to counteract osteosarcopenia. This intervention has been shown to facilitate the regaining of functional independence, improve physical and emotional well-being, and enhance survival through increased muscle mass, reduced fat mass, and improved bone density in breast cancer survivors (BCSs) [4]. Furthermore, engagement in physical activity (PA), particularly strength training, has been demonstrated to reduce mortality risk by 40% through a range of biological mechanisms [5].

Notwithstanding the proven benefits of physical activity for cancer patients, only 8% of patients achieve the recommended 150 min per week of moderate-to-vigorous intensity PA. The majority of these patients remain sedentary and unaware of the potential benefits of exercise for their condition [6,7]. Adherence to PA programs represents a pivotal challenge within the field of oncology, influenced by factors such as motivation, psychological support, and accessibility [8,9]. The concept of adherence to PA programs can be multifaceted, encompassing various dimensions such as attendance, duration, intensity, and consistency over time [10]. 

The creation of adherence to concurrent training for BCS is a particularly challenging endeavor, due to the existence of barriers to the adoption and execution of exercise regimens, which are commonly referred to as oncological rehabilitation [7]. Walking, the most common exercise modality among BCS [2], is insufficient to mitigate treatment side effects. Conversely, concurrent training offers comprehensive benefits, addressing both musculoskeletal and cardiovascular health [4]. Supervised training programs are among the most effective strategies to improve adherence and maximize these benefits, as they provide structured support and tailored guidance [2].

The present study has been designed to evaluate the adherence to moderate and vigorous PA in individuals diagnosed with BCS following a 12-session (6-week) supervised concurrent training intervention. Specifically, the study seeks to determine the adherence rates at three months post-intervention. While the extant literature emphasizes the benefits of PA, there is a paucity of consistent evidence on strategies to sustain adherence in individuals diagnosed with BCS. The present study aims to address this lacuna by assessing the effectiveness of a structured and supervised concurrent training program to promote long-term adherence.

## 2. Materials and Methods

### 2.1. Study Design and Participants

This prospective, multicenter, randomized, parallel-controlled pilot study evaluated the effects of a PA program on BCSs using a case–control design. The study was conducted in 2018 at referral hospitals. Women aged 18 to 65 years, diagnosed with stage I–IIIA breast cancer, free of active disease, and not undergoing chemotherapy were eligible to participate. Exclusion criteria encompassed the presence of chronic conditions that could impede participation in physical activity, significant surgical sequelae, severe lymphedema (>2 degrees), or adherence to the World Health Organization (WHO) recommendation of more than 150 min of moderate or vigorous physical activity per week.

**Figure 1:**
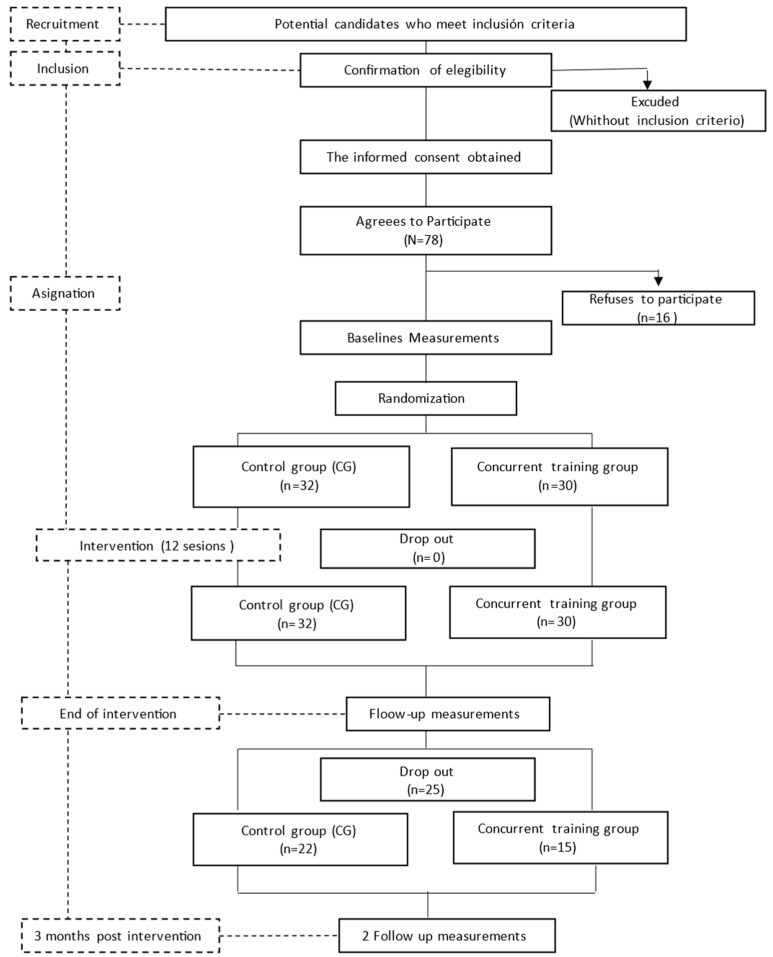
Flow diagram of the study from recruitment to the end of the intervention.

Participants were randomly assigned to groups using a stratified randomization method to ensure balanced allocation based on age and baseline PA levels. Outcome assessors were blinded to group assignments to minimize bias [2]. Initially, 78 eligible women were recruited; however, 16 of these subjects declined to participate, resulting in a final sample of 62 subjects (32 in the intervention group [IG] and 30 in the control group [CG]). It is evident from Figure 1 that all participants completed the intervention and the initial set of questionnaires. At the three-month follow-up, 25 participants were lost to follow-up, leaving 37 participants for final analysis (15 in the IG, with a mean age of 53.14 ± 7.41 years, and 22 in the CG, with a mean age of 53.07 ± 7.43 years). The sample size was determined based on the availability of eligible participants who had undergone treatment at the collaborating hospitals, rather than through a formal power analysis. 

The study participants were provided with comprehensive information regarding its objectives, procedures, and potential risks. Written informed consent was obtained prior to inclusion in the study, which adhered to the principles outlined in the Declaration of Helsinki. The study received approval from the Ethics Committee of the Valencia Oncology Institute and La Fe Hospital (protocol code 2018/0009; approval date: 18 April 2018). Furthermore, the study was retrospectively registered at ClinicalTrials.gov (Identifier: NCT06771635), ensuring transparency and compliance with international standards for clinical research.

The study design took into account potential sources of bias, including dropout rates and retrospective registration, and implemented measures such as stratified randomization and supervised intervention protocols to enhance validity. This robust methodological framework provides a reliable basis for evaluating the effects of the PA program on adherence and outcomes in BCS.

### 2.2. Intervention and Data Collection

The present study concentrated on the evaluation of adherence to moderate-to-vigorous PA in BCSs for three months following the conclusion of a 6-week supervised concurrent training program. The intervention and data collection processes are structured around the different stages of the program.

### 2.3. Program Chronology and Execution

The program was conducted in distinct stages, including assessment, education, intervention, and follow-up phases (see Table 1). This structured approach ensured systematic data collection and effective delivery of the intervention.

- Baseline stage: The baseline stage involved oncologists conducting preliminary evaluations during the first session, encompassing anthropometric measurements (i.e., weight, height, and BMI calculation using the formula [weight (kg)/height (m^2^)], quality of life (QOL) assessment, and PA measurement employing the Global Physical Activity Questionnaire (GPAQ).
- Education Stage: During the second session, both groups participated in a joint talk led by nutritionists on healthy habits and nutrition.
- Intervention Stage: The IG underwent a supervised training program that included 12 sessions of concurrent exercise (see Table 2). Sessions were interspersed to include resistance, cardiovascular, and neuromotor training.
- Evaluation Stage: Participants underwent the same assessments as at baseline three months after completion of the intervention phase.

### 2.4. Physical Activity Intervention

The concurrent training program was designed to address both the physical and psychological needs of BCSs, combining resistance, cardiovascular, and neuromotor training modalities to address multiple aspects of physical function [3,5]. The intervention consisted of twelve supervised sessions, distributed as two 1 h sessions per week over 6 weeks, with the three training modalities alternated to ensure variety and sustain participant motivation (see Table 2). The rationale behind this approach was to prevent monotony, enhance engagement, and promote adherence to the program.

The supervision of all sessions by certified exercise professionals was of paramount importance. The professionals were responsible for ensuring that the correct technique was employed by participants, providing real-time feedback, and adapting exercises to the capabilities of each individual. This supervision was essential for minimizing the risk of injury, fostering a supportive environment, and tailoring progression based on individual progress. The program’s comprehensive benefits encompassed physical enhancements such as increased muscular strength, endurance, coordination, cardiorespiratory fitness, and flexibility. On a psychological level, the program aimed to enhance self-efficacy, alleviate stress, and foster emotional well-being through enjoyable and supportive group interactions.

Resistance Training: Resistance training sessions were structured in the form of a circuit, with rest periods exceeding 30 s between exercises to ensure adequate recovery and safety. Each session comprised three sets of 8 to 12 repetitions per exercise, targeting major muscle groups such as the quadriceps, chest, back, and shoulders. The exercise prescription was individualized and based on a dynamic strength test conducted at the commencement of the study. This test determined the maximum weight that could be lifted for seven repetitions, providing an estimate of the one-repetition maximum (RM) [11]. Initial loads were set at 60% of the one-repetition maximum, a strategy that was intended to promote strength adaptations while minimizing the risk of overexertion.

The program was designed to incorporate progressive overload, with the working weight increasing in steps as the participants demonstrated their ability to complete 12 repetitions. This was increased to 70% of the RM possible, and then to 80% of the RM as strength improved. However, in order to prioritize safety and adherence, loads were not increased beyond 80% of the RM [12,13,14]. The program incorporated a range of exercises, including sumo squats, chest presses, lunges, seated rows, quadriceps curls, and lat pulldowns, providing a comprehensive and balanced approach to developing muscular strength.

Cardiovascular Training: The cardiovascular training program was designed to enhance the aerobic capacity and cardiovascular health of the participants. The participants utilized treadmills, stationary bicycles, or elliptical trainers, with the intensity of their exertion regulated using heart rate monitors. During the sessions, the participants maintained their heart rate at a level between 60 and 75% of their maximum heart rate (HRmax), which was calculated using the following formula: HRmax = 208.75 − (0.73 × age) [15,16,17,18]. The duration of these sessions ranged from 25 to 50 min, with an incremental increase in the program’s duration to align with the participants’ observed improvements in fitness. This gradual progression was theorized to facilitate cardiorespiratory adaptations while maintaining a manageable workload.

Neuromotor Training: Neuromotor training sessions emphasize coordination, balance, and flexibility, which are essential for maintaining functional independence and reducing fall risk in BCS. Sessions incorporated choreographed movements synchronized with music to create an engaging and enjoyable atmosphere, fostering adherence. Stretching exercises were incorporated to improve flexibility and joint mobility, enhancing participants’ range of motion and functional capacity. The intensity of neuromotor training was maintained at a low level, with heart rates kept below 60% of maximum heart rate, as measured using heart rate monitoring devices [19,20,21,22]. This low-intensity approach was found to facilitate recovery, minimize physical strain, and offer psychological benefits through relaxation and stress reduction.

### 2.5. Measurement of Adherence to Physical Activity

The present study utilized the GPAQ [23], a standardized instrument developed by the WHO to evaluate PA across diverse domains and intensities. The GPAQ comprises 16 items; however, this study concentrated specifically on those items pertaining to minutes per day of PA and sedentary behavior. Participants completed the GPAQ at two distinct points: initially before the commencement of the intervention, and subsequently three months after its conclusion. This approach enabled a comprehensive evaluation of the intervention’s sustainability, as well as any alterations in PA levels and sedentary behavior that may have occurred.

In order to facilitate participation, the GPAQ questionnaire was distributed via postal delivery, with several flexible options for participants to return it to the study center, complete it via a telephone interview, or attend in person. Personalized reminders and support were provided to ensure the timely and accurate completion of the questionnaire, and to minimize barriers and dropout rates. This methodological approach ensured the collection of high-quality data while addressing individual needs.

The GPAQ employs a three-domain approach to evaluate PA: work-related PA, encompassing occupational activities of moderate or vigorous intensity; recreational PA, comprising leisure-time pursuits such as exercise, sports, or fitness regimens; and travel-related PA, encompassing activities like walking or cycling for transportation. The intensity of PA is categorized as follows: moderate activities require noticeable physical effort and result in a slight increase in breathing or heart rate; vigorous activities demand significant effort and lead to substantial increases in breathing and heart rate. Sedentary behavior is measured by total daily sitting or reclining time, with sleep time excluded, and is used as an indicator of a sedentary lifestyle.

In this particular instance, adherence to PA is understood to denote an increase in the time that the subject spends engaging in PA at a moderate and/or vigorous intensity. In this study, the minutes of moderate and vigorous PA were disaggregated based on their respective domains of origin, namely work-related and recreational activities. Thereafter, the moderate and vigorous PA minutes from both domains were combined to calculate the moderate-to-vigorous PA (MVPA). Consequently, all PA minutes across domains, with the exception of sedentary behavior, were aggregated to provide a comprehensive measure of the participants’ overall activity levels. Sedentary behavior was analyzed independently to evaluate changes in daily sitting time.

### 2.6. Statistical Analysis

The continuous variables were expressed as the mean ± standard deviation (SD), while the categorical variables were presented as frequencies and percentages (%). The statistical analyses were conducted using SPSS version 28 (IBM Inc., Chicago, IL, USA). A *p*-value < 0.05 was considered to be statistically significant for all tests.

In order to ascertain the normality of continuous variables, the Kolmogorov–Smirnov test was employed (for *n* < 50), with homoscedasticity being evaluated using the Levene test. Differences in sociodemographic characteristics and baseline breast cancer-related variables between groups (IG vs. CG) were assessed using the chi-square test (χ^2^). 

The differences in continuous variables between the IG and the CG were analyzed using a univariate test, with the group to which each participant belonged being treated as a fixed factor. To explore the interaction effects of the intervention over time, a repeated-measures analysis of variance (ANOVA) was performed, with the group factor (IG and CG) included to examine time × group interactions on adherence to physical activity three months post-intervention.

The magnitude of observed effects was quantified using partial eta squared (η^2^*p*). However, it should be noted that partial eta squared may overestimate effect sizes. Therefore, in order to ensure a more accurate interpretation, the guidelines proposed by Ferguson [24] were followed: no effect (0 ≤ η^2^*p* < 0.05), minimal effect (0.05 ≤ η^2^*p* < 0.26), moderate effect (0.26 ≤ η^2^*p* < 0.64), and strong effect (η^2^*p* ≥ 0.64).

## 3. Results

As illustrated in Table 3, the initial study of the 37 BCS revealed that blood pressure data and anthropometric characteristics were homogeneous between groups (IG vs. CG) (*p* > 0.05).

As illustrated in Table 4, the sample’s sociodemographic data reveal no significant disparities in the percentage of BCS across the various variables examined (*p* > 0.05).

As illustrated in Table 5, the following conclusions may be drawn from the data concerning PA outcomes and sedentary behavior at baseline and three months post-intervention for the IG and CG. 

### 3.1. Travel to and from Places PA

At the three-month follow-up, the IG demonstrated a significant increase in travel-related PA, from 26.14 ± 24.20 to 80.68 ± 51.51 min/day, while the CG exhibited a modest rise, from 31.00 ± 32.30 to 54.67 ± 77.36 min/day. No significant time × group interaction effect was observed (*p* = 0.192, η^2^ = 0.050).

### 3.2. PA at Work

At the three-month follow-up, the IG demonstrated a marked increase in moderate physical activity during work hours, from 18.14 ± 58.94 to 77.68 ± 120.51 min per day, while the CG exhibited a decline, from 19.00 ± 62.23 to 8.00 ± 21.11 min per day. A significant time × group interaction effect was observed (*p* = 0.085, η^2^ = 0.082). In the IG, there was a significant increase in vigorous PA at work, from 0.68 ± 3.20 to 7.50 ± 26.08 min/day (*p* < 0.05), while no significant change was observed in the CG (0 to 0.13 ± 0.52 min/day). The IG also demonstrated an enhancement in total PA at work, which includes both moderate and vigorous activities, with a significant increase from 18.82 ± 60.60 to 85.18 ± 122.87 min/day. Conversely, the CG exhibited a decline, from 19.00 ± 62.23 to 8.13 ± 21.06 min/day. Interaction effects were not significant for either vigorous PA or MVPA at work.

### 3.3. Recreational PA

Moderate PA in the IG exhibited a significant increase, from 7.05 ± 12.31 to 66.82 ± 42.80 min/day, while the CG demonstrated a comparatively modest rise, from 10.00 ± 21.04 to 28.80 ± 39.52 min/day. A significant increase in vigorous recreational PA was observed in the IG, from 0 to 20.23 ± 33.68 min/day, while the CG exhibited a modest rise, from 1.67 ± 6.45 to 4.07 ± 11.96 min/day. The IG demonstrated a substantial enhancement in total recreational PA (moderate + vigorous), escalating from 7.05 ± 12.31 to 87.04 ± 55.35 min/day, while the CG exhibited a more modest rise, from 11.67 ± 21.19 to 32.87 ± 43.14 min/day. A significant time × group interaction effect was observed for recreational MVPA (*p* = 0.001, η^2^ = 0.254), but not for vigorous PA.

### 3.4. Combined PA Outcomes (Work + Recreational)

At the three-month follow-up, the IG demonstrated a substantial increase in combined moderate physical activity, with a rise from 25.18 ± 57.94 to 144.50 ± 136.64 min per day. In contrast, the CG exhibited a modest rise, from 29.00 ± 62.51 to 36.80 ± 45.03 min per day. Significant increases in vigorous PA were also observed in the IG, from 0.68 ± 3.20 to 27.73 ± 49.39 min/day, while the CG demonstrated a modest rise, from 1.67 ± 6.45 to 4.20 ± 11.92 min/day. The IG demonstrated a substantial enhancement in total combined PA (moderate + vigorous), escalating from 25.86 ± 59.55 to 167.72 ± 84.81 min/day, while the CG exhibited negligible changes, fluctuating from 172.23 ± 139.50 to 41.00 ± 47.40 min/day. A significant time x group interaction effect was observed for total combined PA (*p* = 0.003, η^2^ = 0.224).

### 3.5. Total PA

The total PA levels, encompassing travel-related, work-related, and recreational PA, exhibited a substantial increase in the IG from 33.18 ± 31.23 to 167.72 ± 84.81 min/day, while the CG demonstrated a modest rise, from 42.67 ± 31.90 to 87.53 ± 93.74 min/day. A significant time × group interaction effect was observed (*p* = 0.006, η^2^ = 0.197).

### 3.6. Sedentary Behavior

The IG demonstrated a significant reduction in sedentary time, from 330.91 ± 179.89 to 220.14 ± 275.58 min/day, while the CG exhibited no substantial changes, exhibiting a slight decrease from 290.00 ± 163.40 to 274.00 ± 158.29 min/day. No statistically significant time × group interaction effect was observed (*p* = 0.228, η^2^ = 0.071).

## 4. Discussion

The objective of this study was to ascertain the degree of adherence to PA in BCSs following a six-week concurrent training intervention, as defined by an increased time spent performing moderate and/or vigorous PA. The findings underscore the efficacy of this intervention in promoting adherence, curtailing sedentary behavior, and augmenting the time spent in moderate and vigorous PA, which are pivotal components of enhanced health outcomes in BCS [25,26].

The IG demonstrated significant improvements in adherence, as reflected by the increased time spent performing moderate and vigorous PA across all domains at three months post-intervention. Statistically significant differences were observed between the IG and the CG in vigorous PA, moderate PA, and combined MVPA for both recreational and work-related activities. These findings are consistent with previous research indicating that supervised, structured training interventions effectively enhance adherence to PA among BCS [12,16]. The positive outcomes observed in the IG underscore the importance of incorporating supervised exercise programs into survivorship care plans to promote long-term engagement in PA.

A pivotal element contributing to these outcomes is the structured nature of the intervention, which furnished participants with explicit guidance, consistent schedules, and professional supervision. Structured programs offer consistency and accountability, which are pivotal in fostering PA adherence, particularly in populations such as BCS, who may encounter physical and psychological barriers to exercise participation [7]. The presence of qualified exercise professionals has been demonstrated to contribute to participants’ confidence and motivation, ensuring adherence to prescribed exercise protocols and facilitating progressive overload safely [19].

Furthermore, social support and group dynamics have been shown to play a critical role in PA adherence among BCS [7,8]. Participants in the IG benefitted from a sense of camaraderie, shared experiences, and mutual encouragement, all of which have been identified as strong predictors of sustained PA engagement [7,12,19]. Exercising in a group setting has been shown to reduce feelings of isolation commonly experienced by BCS and to provide a sense of accountability and emotional support, both of which are vital for maintaining long-term adherence [16]. Studies have shown that individuals who engage in group-based PA programs report higher levels of motivation, enjoyment, and commitment compared to those exercising individually [16].

Another critical element influencing adherence is the psychological benefits derived from PA participation. Evidence suggests that regular PA in BCS is associated with reductions in cancer-related fatigue, anxiety, and depression, which in turn positively reinforce adherence to exercise regimens [27,28,29]. Participants in the IG may have experienced improvements in overall well-being, leading to a greater commitment to maintaining an active lifestyle even beyond the intervention period. Furthermore, the positive reinforcement from experiencing tangible health benefits, such as increased energy levels and improved physical function, may have further contributed to adherence [30].

Notwithstanding the encouraging outcomes of this study, it is imperative to recognize the challenges associated with adherence to PA among individuals diagnosed with BCS. These challenges encompass a range of individual-level factors, including time constraints, physical limitations arising from cancer treatments, and competing responsibilities. The integration of personalized interventions, encompassing flexible exercise options such as home-based programs and digital health tools, holds considerable potential in addressing these barriers and fostering long-term adherence to PA, extending beyond the scope of supervised interventions [27,31].

This study has confirmed the established correlation between PA and positive health outcomes in BCSs. The analysis indicates that consistent participation in moderate-to-vigorous PA is associated with a reduced risk of breast cancer recurrence, enhanced survival rates, and improved quality of life [30,32]. The findings underscore the importance of promoting adherence to PA through structured and supervised programs as a pivotal component of post-treatment care for BCS.

The present study has several limitations that should be taken into account when interpreting the results. First, the loss of data from participants who did not complete the three-month follow-up may have introduced bias and reduced the statistical power of the results. Secondly, the sample size was determined based on the availability of eligible participants who had completed treatment at the collaborating hospitals, rather than through a formal power analysis. Although this approach allowed for a comprehensive assessment of adherence to physical activity following a concurrent exercise intervention, it may limit the statistical robustness and generalizability of the findings. Thirdly, although the six-week intervention was sufficient to produce positive changes, a longer intervention period may have produced more sustained effects. In addition, the study was conducted in two hospitals in Spain, which may limit the generalizability of the results to other populations. Finally, the potential influence of dietary habits and psychosocial support on the results was not taken into account, which may have introduced bias.

The results of this study have important practical implications for healthcare providers, patients, and policymakers. Healthcare providers can use these findings to advocate for the integration of structured exercise programs as a standard component of survivorship care, ensuring that breast cancer survivors receive evidence-based support to improve their adherence to physical activity. Patients can benefit from increased awareness of the importance of participating in supervised exercise programs, which have been shown to reduce sedentary time and improve long-term health outcomes. In addition, policymakers should consider incorporating similar exercise interventions into national and institutional survivorship guidelines to promote adherence and support the well-being of breast cancer survivors more widely.

Despite these limitations, the study has several strengths. The concurrent training intervention, which combined strength, aerobic, and neuromotor exercises, provided a comprehensive approach rarely implemented in BCS populations. Collaboration with two leading hospitals ensured methodological rigor and a representative sample. The program was designed and overseen by professionals specializing in physical activity and sports sciences, thus ensuring safe and effective implementation tailored to the needs of the BCS population. Moreover, the dropout rate in this study may have influenced the generalizability of the findings, a challenge commonly observed in PA interventions for cancer survivors [11].

It is recommended that future research extend follow-up periods in order to evaluate the long-term sustainability of PA adherence and sedentary behavior reduction in BCS. The development of personalized exercise interventions that address individual physical and psychological barriers, and the integration of technology such as wearable devices and telehealth, has the potential to enhance engagement and motivation. Comparative studies with other PA modalities, such as yoga or pilates, may help identify the most effective strategies for improving adherence and health outcomes. Furthermore, the inclusion of a more diverse population in future studies is recommended, with the aim of enhancing the generalizability of findings across a range of demographic and cultural contexts.

## 5. Conclusions

In conclusion, the findings of this study indicate that a supervised six-week concurrent training intervention, which included strength, aerobic, and neuromotor exercises, effectively increases adherence to PA in BCSs three months post-intervention. The intervention, meticulously planned and overseen by exercise specialists, yielded substantial enhancements in PA adherence across diverse intensity levels, particularly in moderate, vigorous, and combined moderate-to-vigorous PA, within both recreational and occupational domains. These outcomes underscore the efficacy of structured concurrent training programs in cultivating enduring exercise behaviors among BCS well beyond the immediate intervention period.

Furthermore, the intervention contributed to a significant reduction in sedentary time, emphasizing its potential to address the sedentary lifestyle commonly observed in this population. The structured and supervised nature of the program is likely to have played a critical role in maintaining adherence, reinforcing the importance of professional guidance and social support in promoting long-term behavioral changes.

## Figures and Tables

**Table 1:** Chronology of physical activity intervention and breast cancer survivor assessment.

Session No.	Type	Responsible	Shares
1	Patient assessment	Oncologist	BMI, QOL, PA
2	Education in healthy habits	Nutritionist	Joint talk (Experimental group + control group)
3–15	Concurrent exercise program	Trainer	
3 months	Evaluation	Oncologist	BMI, QOL, PA

BMI: body mass index; QOL: quality of life; PA: physical activity.

**Table 2:** Physical activity intervention planning.

Type	Session No.	Load	Exercises
Resistance Training	1, 4, 7, 10	3 series; 8–12 repetitions; 60–80% RM	Sumo Squat, Chest Press, Lunge, Seated Row, Quadriceps Curl, Lat Pulldown
Cardiovascular Training	2, 5, 8, 11	25–50 min; 60–75% HRmax	Treadmill, Bicycle (Cycloergometer), Elliptical
Neuromotor Training	3, 6, 9, 12	≤60% HRmax	Choreographies with musical support and stretching

RM: maximum repetition; HRmax: maximum heart rate.

**Table 3:** Blood pressure and anthropometric data at baseline of BCS by study group.

	GI (*N* = 15)	GC *(N* = 22)	*p*-Value
Age (years)	53.14 ± 7.41	53.07 ± 7.43	0.978
Weight (kg)	68.31 ± 11.98	62.98 ± 9.72	0.165
Height (m)	1.62 ± 0.06	1.61 ± 0.06	0.674
BMI	26.13 ± 3.87	24.20 ± 3.10	0.120

Data are expressed as mean ± standard deviation. *p*-value: Determined as a one-factor ANOVA.

**Table 4:** Sociodemographic characteristics at baseline of BCS by study group.

	GI (*N* = 15)	GC (*N* = 22)	*p*-Value
Marital status			
Married	68.2	66.7	0.419
Single	22.7	33.3	
Widow	9.1	0.0	
Level of Education			
No education	0.0	13.3	0.353
Primary education	27.3	26.7	
Secondary education	45.5	33.3	
University or higher	27.3	26.7	
Employment status			
Employee	63.6	40.0	0.100
Housewife	31.8	26.7	
Unemployed	0.0	20.0	
Disability person	4.5	13.3	
Alcohol consumption (Yes)	9.1	0.0	0.230
Tobacco Consumption (Yes)	9.1	13.3	0.683
Obesity (Yes)	4.5	0.0	0.403
Hypertension (Yes)	13.6	13.3	0.979
Dyslipemia (Yes)	22.7	13.3	0.474
Diabetes Mellitus (Yes)	0.0	0.0	1.000
Osteoporosis (Yes)	13.6	0.0	0.136
Heart disease (Yes)	0.0	0.0	1.000
Pulmonary disease (Yes)	4.5	6.7	0.779
Thyroid (Yes)	9.1	13.3	0.683
Anxiety/depression (Yes)	9.1	0.0	0.230

Data expressed in % of cases. *p*-value: Determined through the Chi-square test.

**Table 5:** Follow-up of physical activity performed by the BCS.

Moment	IG (*N* = 22)	GC (*N* = 15)	*p*	η^2^
Travel to and from places PA (minutes/day)				
Baseline	26.14 ± 24.20	31.00 ± 32.30	0.192	0.050
3 months	80.68 ± 51.51	54.67 ± 77.36		
Moderate Intensity PA at Work (minutes/day)				
Baseline	18.14 ± 58.94	19.00 ± 62.23	0.085	0.082
3 months	77.68 ± 120.51 *$	8.00 ± 21.11		
Vigorous Intensity PA at Work (minutes/day)				
Baseline	0.68 ± 3.20	0 ± 0	0.337	0.026
3 months	7.50 ± 26.08 *	0.13 ± 0.52		
MVPA at Work (minutes/day)				
Baseline	18.82 ± 60.60	19.00 ± 62.23	0.053	0.123
3 months	85.18 ± 122.87 *$	8.13 ± 21.06		
Recreational Moderate Intensity PA (minutes/day)				
Baseline	7.05 ± 12.31	10.00 ± 21.04	0.006	0.200
3 months	66.82 ± 42.80 *	28.80 ± 39.52		
Recreational Vigorous Intensity PA (minutes/day)				
Baseline	0 ± 0	1.67 ± 6.45	0.061	0.097
3 months	20.23 ± 33.68 *$	4.07 ± 11.96		
Recreational MVPA (minutes/day)				
Baseline	7.05 ± 12.31	11.67 ± 21.19	0.001	0.254
3 months	87.04 ± 55.35 *$	32.87 ± 43.14		
Recreational + Work Moderate Intensity PA (minutes/day)				
Baseline	25.18 ± 57.94	29.00 ± 62.51	0.016	0.155
3 months	144.50 ± 136.64 *$	36.80 ± 45.03		
Recreational + Work Vigorous Intensity PA (minutes/day)				
Baseline	0.68 ± 3.20	1.67 ± 6.45	0.073	0.089
3 months	27.73 ± 49.39 *$	4.20 ± 11.92		
Recreational + Work Total PA (minutes/day)				
Baseline	25.86 ± 59.55	172.23 ± 139.50	0.003	0.224
3 months	30.67 ± 62.05 *$	41.00 ± 47.40		
Total PA (minutes/day)				
Baseline	33.18 ± 31.23	42.67 ± 31.90	0.006	0.197
3 months	167.72 ± 84.81 *$	87.53 ± 93.74		
Sedentary (minutes/day)				
Baseline	330.91 ± 179.89	290.00 ± 163.40	0.228	0.071
3 months	220.14 ± 275.58 $	274.00 ± 158.29 $		

Data presented as mean ± standard deviation. PA: physical activity; MVPA: moderate-to-vigorous physical activity; *p*: interaction effects of time × group; *: significant differences between groups (IG vs. CG); $: significant differences within groups over time (baseline vs. 3 months).

## Data Availability

The data of the research is availed on the tables of the manuscript or on request from the corresponding author.
